# Latent profiles of attitudes toward ageing during the nursing home “early transition period” and its correlation with quality of life

**DOI:** 10.1186/s12877-026-07007-7

**Published:** 2026-02-07

**Authors:** Luyi Xu, Tingting Lin, Zheng Wang, Siyi Su, Jufang Li, Ping Li

**Affiliations:** School of Nursing, Wenzhou Medical University, Wenzhou, Zhejiang China

**Keywords:** Attitudes toward ageing, Quality of life, Self-efficacy, Nursing home, Latent profile analysis, Mediation analysis

## Abstract

**Purpose:**

This study aimed to identify the heterogeneity of attitudes toward ageing among older adults in the “early transition period” (the initial 2–4 weeks after nursing homes transition from home to nursing homes). and the mediation effect of self-efficacy between attitudes toward ageing and quality of life (QoL).

**Method:**

A total of 300 older adults were enrolled from October 2023 to May 2024. Participants completed the General Information Questionnaire, the Attitudes to Ageing Questionnaire (AAQ), the World Health Organization Quality of Life-Brief (WHOQOL-BREF), and the General Self-Efficacy Scale (GSES). Latent profile analysis (LPA), R3STEP methods, BCH methods, and mediation analysis were conducted to analyze the data.

**Results:**

LPA categorized the attitudes toward ageing into three profiles: most negative (18.333%), moderately negative (64.000%), and positive (17.667%). Attitudes toward ageing profiles were associated with the following factors: age, pension, number of children, number of chronic diseases, ADL, willingness to reside in nursing homes, and social isolation. Self-efficacy partially mediates between attitudes toward ageing and the three dimensions of QoL (physical health, psychological health, and environmental health).

**Conclusions:**

Older adults during the “early transition period” had negative attitudes toward ageing. It may be related to the Chinese traditional interpersonal communication mode, family culture, and various maladaptive problems. Older adults who have two or more children, chronic diseases, no pension, moderate to severe dependency, involuntary admission to nursing homes, and social isolation are associated with more negative attitudes toward ageing. Mediation analysis reminds that self-efficacy can be used as intervention targets to improve the QoL.

**Supplementary Information:**

The online version contains supplementary material available at 10.1186/s12877-026-07007-7.

## Introduction

In China, with the rapid growth of the older adult population, changes in family structure, improved medical care, and longer life expectancy, the traditional family model of older adult care can no longer effectively meet the care needs of older adults, and more and more older adults are opting for institutional care [1]. However, during the transition from home to nursing homes, older adults often experience a “chaotic period” (i.e.,“early transition period”) [2, 3]. The “early transition period” specifically refers to the initial 2–4 weeks after older adults transition from their family home to a residential care facility [3, 4]. During this period, older adults leave homes they have lived in for decades, which means losing familiar surroundings, independent living, part of their social circle, and even a sense of “control” over their own lives [5]. At this time, their hearts are filled with conflict, unease, and a sense of loss, while their behavior exhibits various forms of maladjustment and negative emotions [2, 3, 5]. This period can lead to self-centred behaviours, weakened social connections, and loneliness, resulting in mental health problems that seriously affect their quality of life [2, 6].

Quality of life (QoL) refers to an individual’s perception of their life’s goals, expectations, standards, and concerns [7]. It has become an important indicator of health under the bio-psycho-social medical model and an important element in evaluating active ageing [8]. Older adults in the “early transition period” suffer changes in their living environment and are prone to various maladjustments [9], which have an impact on their physical and mental health, as well as their QoL and longevity. Therefore, it is important to pay attention to the physical and mental health and QoL of older adults during the “early transition period.”

In China, nursing homes are generally categorized into three types: publicly built and publicly operated (public-public), publicly built and privately operated (public-private), and privately built and privately operated (private-private) [10].

Public-public nursing homes are built and operated by the government. They primarily serve older adults in special hardship (such as those without children, income, or ability to work) and low-income older adults with functional impairments [10]. These institutions charge very little or no fees and provide basic life care, such as meals, cleaning, and assistance with daily living. Their services are singular in nature, offering only basic social stimulation through simple recreational activities like group walks or board and card games [9]. As most residents are vulnerable older adults without immediate family, there is minimal need for communication with relatives, resulting in limited family involvement [11].

Public-private nursing homes cater to the general population of older adults, charging moderate fees. They prioritize admitting those with functional impairments, partial disabilities, or cognitive disorders (such as dementia), while also accommodating the needs of some seniors who are largely self-reliant [10]. The scope of care includes daily living assistance, rehabilitation, and nursing. Furthermore, due to the involvement of non-governmental entities, some facilities introduce specialized services like dementia-specific care or traditional Chinese medicine therapy [10]. Communication with family members is more frequent and flexible, utilizing methods like WeChat and phone calls to provide updates.

Privately-built and privately-operated nursing homes’ care services vary significantly: mid- and low-end nursing homes focus on basic daily living assistance, whereas high-end ones offer tailored, personalized services. Some high-end facilities employ full-time rehabilitation therapists and recreational specialists to run cognitive-training programs (e.g., number-sequence games, art therapy) and regularly organize themed events such as birthday parties and holiday celebrations; they even bring in outside resources like university courses and volunteer services [10].

Driven by ample local private capital and strong investor interest, the government has encouraged private investment in nursing homes through subsidies and policy guidance. Consequently, publicly-run and publicly-operated nursing homes are notably scarce in our area. The sample for this survey was therefore primarily drawn from two categories of institutions: publicly-built, privately-operated and public-private and private-private nursing homes.

In summary, the service priority for nursing homes in China remains centered on providing daily living assistance for older adults, with only a limited number of high-end facilities capable of addressing their diverse needs. Furthermore, previous studies have also indicated that nursing homes in China rarely pay adequate attention to the psychological well-being of older residents [12]. The mental health status of older adults in such settings is often suboptimal, with a majority experiencing mild or more severe psychological issues [6]. Therefore, focusing on their psychological needs and identifying effective intervention pathways to address these challenges are crucial for helping them adapt successfully to institutional life and, ultimately, for enhancing their overall QoL.

Attitudes toward ageing is a psychological variable to measure older adults’ physical and mental well-being. It refers to people’s experience and appraisal of the ageing process, which can be divided into positive and negative experiences [13]. Negative attitudes toward ageing can lead to negative mental and physical conditions in older people [14]. Positive attitudes toward ageing can improve emotional function and mental health in older adults, which is an important factor in promoting healthy ageing and has important significance for the implementation of a healthy ageing strategy [15]. Older adults in Chinese nursing homes have medium to low attitudes toward ageing [16]. Older adults still in the “early transition period”, influenced by environmental changes and physical health conditions, are more likely to have negative attitudes toward ageing. Previous studies have mostly focused on the relationship between the total score of attitudes toward ageing and individual variables, ignoring individual variations and potential variability. Latent profile analysis (LPA) is a person-centred modelling method that can classify heterogeneous samples into more meaningful subgroups [17]. Therefore, this study conducted an LPA of older adults’ attitudes toward ageing during “early transitionperiod” in nursing homes, identifying potential subgroups and their characteristics to provide a reference for the formulation of targeted intervention measures.

Previous research has found that an individual’s attitudes toward ageing play an important role in predicting the QoL of old adults [18]. The positive attitude toward ageing is positively correlated with better physical health, whereas a negative attitude can exacerbate physical decline [19, 20]. Furthermore, a longitudinal study demonstrated that attitudes toward ageing significantly impact the physical and psychological well-being, as well as the behaviors, of older adults, thereby ultimately diminishing their QoL [21]. However, the relationship between attitudes toward ageing and QoL of older adults in nursing homes, especially during the “early transition period,” requires further research.

Self-efficacy, an individual’s perceived ability to do a certain task, is a cognitive process that affects the regulation of motivation and mental processes throughout specific behavioural processes [22]. A previous study on community-dwelling older adults revealed that expectations regarding ageing, which represent a core dimension of positive attitudes toward ageing, can indirectly influence QoL through self-efficacy [23]. Specifically, among older adults, a positive attitude toward ageing may motivate individuals to engage more proactively in social activities and learn new skills (e.g., using smart devices). These successful experiences reinforce their self-efficacy [23]. Conversely, those with low self-efficacy, often influenced by negative perceptions of ageing, may withdraw from social interactions and reduce health management behaviors, ultimately leading to a decline in QoL [23, 24]. However, this effect has not yet been verified among older adults during the “early transition period” and still requires further exploration. These older adults, affected by drastic environmental changes, often hold relatively negative attitudes toward ageing, which subsequently impacts their QoL. If self-efficacy plays a role in this pathway, it could serve as a potential target for future interventions.

This study’s theoretical framework is the transactional models of stress and coping [25]. This theory states that in the case of continuous pressure, individuals will make a cognitive evaluation of the current situation, and then they can actively adjust their cognitive level to resist the threat of pressure and effectively cope with current challenges. In the face of pressure from various aspects such as environmental change and weakened social connections, older adults newly admitted to a nursing home will re-evaluate their physical and mental health, produce different experiences and evaluations (attitudes toward ageing) from their old age before they stay in the nursing home, and then actively adjust their cognitive level (self-efficacy) to resist the threat of pressure, thereby improving the QoL. Based on the above theories, this study proposes the following hypotheses:

H1 Attitudes toward ageing are characterized by population heterogeneity.

H2 Self-efficacy mediates the relationship between attitudes toward ageing and QoL.

## Methods

### Design and participant

This was a cross-sectional study. Purposive sampling was adopted to select nursing homes distributed across different urban and suburban districts in Wenzhou, with a total of 15 nursing homes ultimately chosen as the research sites.

The purposeful sampling method was adopted to select the newly admitted older adults in nursing homes as the research participants from October 2023 to May 2024, and a questionnaire survey was conducted on the population meeting the inclusion criteria of this study. The inclusion criteria are as follows: (1) aged 60 years old or older; (2) resided in a nursing home for 2–4 weeks; (3) able to communicate and complete questionnaires (4) volunteer to participate in this survey. The exclusion criteria are as follows: (1) moved in from another nursing home; (2) in the acute onset or terminal stage of disease; (3) presence of cognitive impairment at any level (Mini-Mental State Examination: illiterate ≤ 17, primary school ≤ 20, junior high school and above ≤ 24) [26]. After distributing the questionnaires, the researcher explained the methods and key points for completing them to the participants. This ensured that they clearly understood the process and could fill out the questionnaires independently based on their actual circumstances. For those who were illiterate or unable to complete the questionnaire independently due to physical reasons, the investigators conducted interview-based surveys using a uniform set of instructions.

The sample size was planned based on dual considerations: model stability for LPA and statistical power for inter-profile comparisons. To address the former, LPA guidelines recommend 300–500 participants [27]. For the latter, an a priori power analysis using G*Power 3.1 indicated that a minimum of 159 participants would be required to detect inter-profile differences (effect size = 0.25, α = 0.05, power = 80%) [28]. In this study, 330 questionnaires were distributed, yielding 300 valid responses after the exclusion of 30 incomplete ones. This final sample size is sufficient to satisfy both criteria.

### Measurement

#### Demographic, ADL, social isolation

Demographic information was collected from participants, including age, sex, marital status, educational level, pension, insurance, willingness to be admitted to nursing homes, number of chronic diseases, and number of children. The Barthel Index was used for ADL assessment in this study [29]. It has five grades: complete independence (100 points), mild dependence (75–95 points), moderate dependence (50–70 points), severe dependence (20–39 points), and complete dependence (< 20 points). The Lubben Social Network Scale-6 (LSNS-6) was developed by Lubben [30] and translated into Chinese by Chang [31] to measure social isolation. It comprises two dimensions, family network and friend network, with a total score of 0–30. The lower scores indicate a higher risk of social isolation. A total score of less than 12 is considered social isolation. The Cronbach’s α of this scale was 0.83 [31]. The Cronbach’s α and McDonald’s Omega coefficients were 0.766 and 0.735 in this study, respectively.

#### Attitudes toward ageing

The Attitudes to Ageing Questionnaire (AAQ) was developed by Laidlaw [13] and translated into Chinese by Huang [32] to measure older adults’ evaluation and perception of the ageing process. The questionnaire has 24 items and covers three dimensions: psychosocial loss, physical change, and psychological growth [13]. All items are self-report, with ratings ranging from 1 to 5, where 1 reflects strong disagreement or not at all true, and 5 reflects strong agreement or extremely true. In this scale, higher scores on the physical change and psychological growth dimensions indicate more positive attitudes toward ageing. Psychosocial loss, however, is a negative dimension, meaning that higher original scores reflect more negative attitudes. To simplify data analysis, the items in this dimension were reverse-scored (e.g., 1→5, 2→4, 3→3, 4→2, 5→1) so that higher final scores would likewise represent more positive attitudes toward ageing, aligning with the direction of the positive dimensions. The total AAQ score ranged from 24 to 120, with 72 as the theoretical midpoint of the scale. Older adults with a score ≥ 72 had positive attitudes toward ageing. After reverse scoring, higher scores on all three AAQ dimensions indicate more positive attitudes toward ageing. This scale has a Cronbach’s α of 0.827 [32]. The Cronbach’s α and McDonald’s Omega coefficients were 0.798 and 0.819 in this study, respectively.

#### Quality of life

The World Health Organization Quality of Life-Brief (WHOQOL-BREF) translated into Chinese by Prof. Fang, was used to evaluate the QoL of older adults in nursing homes [33, 34]. This scale consists of 26 items, with a 5-point Likert scale employed for all items. The scale includes 4 dimensions, including physical health (7 items), psychological health (6 items), social relationships (3 items), and environmental health (8 items). Of these, two are stand-alone items that were not included in this study. The score of each dimension is calculated by multiplying the average score of the items in that dimension by four. Higher scores are associated with a better quality of life. This scale was developed from WHOQOL-100 (Cronbach’s α was 0.794), and there is a high correlation between the two scales [35]. The Cronbach’s α and McDonald’s Omega coefficients were 0.906 and 0.910 in this study, respectively.

#### Self-efficacy

The General Self-efficacy Scale (GSES) was developed by Schwarzer [36] and translated into Chinese by Wang [37]. It has 10 items with a Likert 4 scale ranging from 10 to 40, with higher scores indicating stronger self-efficacy. The scale has three grades: low (10–20 points), medium (21–30 points), and high (31–40 points). This scale has a Cronbach’s α of 0.87 [37]. The Cronbach’s α and McDonald’s Omega coefficients were 0.917 and 0.918 in this study, respectively.

### Statistical analysis

First, Harman’s one-way factor model was used to verify the potential existence of common method variance (CMV). Skewness and kurtosis are used to evaluate the normality of continuous variables. If kurtosis is less than 10 and skewness is less than 3, the data can be considered normal [38].

Second, Mplus 8.3 was used to conduct the LPA and identify the attitudes toward ageing subgroups using the three AAQ dimensions. In order to base the profile classification squarely on the three core dimensions of attitudes toward aging (psychosocial loss, physical change, and psychological growth), the LPA analysis utilized their dimension scores. Due to the consistent structure across dimensions—each comprising 8 items with a total score range of 8 to 40—no standardization was applied prior to analysis. A series of model fit indices were used to assess model’s goodness, including Akaike’s information criterion (AIC), the Bayesian information criterion (BIC), the adjusted BIC (aBIC), and Entropy [27]. Lower values in the AIC, BIC, and aBIC represented a better model fit [27]. An entropy value closer to 1 indicates more accurate classification, with a value of ≥ 0.80 suggesting that the model’s classification accuracy reaches approximately 90% [39]. The Lo-Mendell-Rubin test (LMR) and the bootstrap likelihood ratio test (BLRT) were used to compare k-class models with k-1 class models. The P-values of LMRT and BLRT are significant, indicating that the k-class model can fit the data better than the k-1 class model [40]. Besides, the number of people in each profile needs to exceed at least 5% of the total study subjects. To ensure the stability of the model solution and avoid local optima, we performed 1000 random starts. An ANOVA was used to examine the differences in attitudes toward ageing among the profiles.

Third, a latent multinomial logistic model that regressed the latent profiles variable on major demographic characteristic was estimated using the robust three-step method (R3STEP) [41, 42]. The BCH command in Mplus was employed to model the outcome variables of self-efficacy and QoL. This analysis compared latent profiles and determined whether each profile significantly differed from other profiles in predicting outcome variables [41].The advantages of R3STEP and BCH are that they enhances statistical power by accounting for classification uncertainty.

Fourth, Pearson correlation analysis was used to determine the relationship between attitudes toward ageing, self-efficacy, and QoL. Then, the four dimensions of QoL were analyzed by univariate factor analysis, and the variables with significant influence on QoL were included in the mediation model as covariables for analysis. Then, the mediating role of self-efficacy between LPA-based attitudes toward ageing profiles (category variables) and QoL (including four dimensions) was estimated using the PROCESS macro (Model 4) of SPSS. In the analysis, the bootstrap procedure was set at 5000 iterations, with Profile 1 designated as the reference group.

## Results

### Common method variance test

In this study, the common method bias test was carried out for self-assessment questionnaires. The results showed that there were 15 factors with characteristic roots greater than one, and the first factor explained 25.605% of the variance (less than the critical value of 40%), indicating no serious common method bias problem in this study.

### Participants’ demographic characteristics, social isolation

In this study, there were 162 female older adults (accounting for 54.0%) and 138 male older adults (accounting for 46.0%). In terms of age stratification, 56 participants were aged 60–74 years (18.67%), 184 were aged 75–89 years (61.33%), and 60 were aged 90 years and above (20.00%). In terms of educational level, 210 participants had an education level of primary school or below (70.00%), 62 had a junior or senior high school education (20.67%), and 28 had a college education or above (9.33%). 207 older adults lived in public-private nursing homes, while 93 older adults lived in private-private nursing homes. Besides, in this study, 240 (80%) older adults had an LSNS-6 score ≤ 12, which indicated social isolation.

### Latent profile analysis of AAQ

The model fit statistics for 2–4 latent profile models are shown in Table 1. We ultimately selected the three-profile model for the following reasons: (1) As the number of latent profiles increased from one to three, the AIC, BIC, and aBIC values decreased significantly, and both the LMR and BLRT tests were statistically significant (*p* < 0.001); (2) The entropy value exceeded 0.80, indicating that the model’s classification accuracy reached 90%; (3) The smallest profile group size accounted for more than 5% of the total sample. Besides, four-profile solution was rejected due to the non-significant LMR test and the very small class size.

**Table 1:** Model fit indices for the compared latent profiles (*n* = 300)

Indices	LPA model		
	2-profile	3-profile	4-profile
Fit statistics			
AIC	845.259	**697.032**	679.044
BIC	882.297	**748.885**	745.712
aBIC	850.583	**704.485**	688.627
Entropy	0.896	**0.906**	0.904
LMR	<0.001	**<0.001**	0.309
BLRT	<0.001	**<0.001**	<0.001
Group size(%)			
C1	247.000 (82.333**%**)	**55.000 (18.333%)**	182.000 (60.667%)
C2	53.000 (17.667**%**)	**192.000 (64.000%)**	58.000 (19.233%)
C3	-	**53.000 (17.667%)**	7.000 (2.333%)
C4	-	-	53.000 (17.667%)

Supplementary materials Figure S1 shows the 3-profile model based on LPA. Profile 1 (*n* = 55, 18.333%) had the lowest scores on all three dimensions compared to the other groups, and the mean total AAQ score (57.364 ± 3.894) was much lower than 72, so it was named “most negative”. The average AAQ score (69.948 ± 2.883) of Profile 2 (*n* = 192, 64.000%) is still below 72, but higher than Profile 1, and is therefore labelled as “moderately negative”. Profile 3 (*n* = 53, 17.667%) is the only group with a mean (83.604 ± 4.325) of more than 72 points and is named “positive”.

The ANOVA and post hoc test (see Table 2) showed that the total AAQ scores of the three profiles and the scores of the three dimensions were significantly different (*p* < 0.05). The mean scores for Profile 1 on psychosocial loss, physical change, and psychological growth were 22.873, 19.927, and 14.564, respectively. The mean scores for Profile 2 on psychosocial loss, physical change, and psychological growth were 26.453, 26.234, and 17.260, respectively. The mean scores for Profile 3 on psychosocial loss, physical change, and psychological growth were 33.528, 31.075, and 19.000, respectively. The **η**^**2**^ of psychosocial loss, physical change, and psychological growth were 0.665, 0.708, and 0.346, respectively. This indicates that profile membership explained 66.5%, 70.8%, and 34.6% of the variance in these respective outcome variables. According to the truncation criteria of **η**^**2**^, all three dimensions showed large effect sizes [43].

**Table 2:** Differences of attitudes toward ageing scores among three different latent profiles

Variables	Profile 1 Mean (SD)	Profile 2 Mean (SD)	Profile 3 Mean (SD)	*p*	η^2^	Post-hoc test
Psychosocial loss	22.873(2.365)^a^	26.453(2.470)^b^	33.528(1.793)^c^	<0.001	0.665	a< b,c; b< c
Physical change	19.927(1.730)^a^	26.234(2.309)^b^	31.075(2.065)^c^	<0.001	0.708	a< b,c; b< c
Psychological growth	14.564(1.371)^a^	17.260(1.753)^b^	19.000(2.594)^c^	<0.001	0.346	a< b,c; b< c

a, b, c showed the results of the post-hoc tests and there is a statistically significant between two. e.g. for Total AAQ ‘a < b, c’ means that Profile 1 has a mean score that is lower than Profile 2 and Profile 3. For those with homogeneity of variance, ANOVA and Bonferroni test were used for difference test and post hoc test. Welch’s test and Tamhane test were used for ‘psychological growth’ without homogeneity of variance for difference test and post hoc test

*Profile 1* “most negative” group, *Profile 2* = “moderately negative” group, *Profile 3* “positive” group

The results of the R3STEP method for predicting variables are shown in Table 3. Compared with the “positive” group, older adults in the “most negative” group were characterized by having no pension, having comorbidities (number of chronic diseases ≥ 2), having two or more children, and a moderate to severe dependence in ADL.

**Table 3:** Results of predictor variables for the robust 3-step approach

Variables	Profile 1 VS Profile 3				Profile 2 VS Profile 3				Profile 1 VS Profile 2			
	B	SE	*P*	OR (95%CI)	B	SE	*P*	OR (95%CI)	B	SE	*P*	OR (95%CI)
Age (Ref. ≥ 90)												
75–89	0.741	1.207	0.539	2.098 (0.197,22.359)	-1.416	0.783	0.071	0.243 (0.052,1.127)	2.157	0.988	**0.029**	8.644 (1.248,59.886)
60–74	-0.011	1.374	0.993	0.989 (0.067,14.608)	-1.825	0.854	**0.033**	0.161 (0.030,0.861)	1.813	1.185	0.126	6.130 (0.601,62.535)
Sex (Ref. Female)												
Male	0.777	0.729	0.287	2.174 (0.521,9.075)	0.070	0.416	0.867	1.072 (0.474,2.424)	0.707	0.689	0.305	2.028 (0.525,7.833)
Marital status (Ref. Married)												
Widowed/Divorced/Single	-0.378	0.712	0.595	0.685 (0.170,2.768)	0.308	0.444	0.489	1.360 (0.569,3.250)	-0.686	0.635	0.280	0.504 (0.145,1.749)
Educational level (Ref. Primary school and below)												
Middle and high school	-0.638	0.752	0.396	0.528 (0.121,2.307)	-0.484	0.409	0.237	0.617 (0.276,1.375)	-0.154	0.718	0.830	0.857 (0.210,3.499)
College and above	0.994	0.979	0.310	2.702 (0.397,18.401)	-0.699	0.622	0.261	0.497 (0.147,1.683)	1.693	0.950	0.075	5.433 (0.844,34.980)
Pension (Ref. Yes)												
No	3.469	0.900	**< 0.001**	32.094 (5.499,187.306)	1.255	0.457	**0.006**	3.509 (1.433,8.594)	2.213	0.853	**0.009**	9.145 (1.718,48.692)
Insurance (Ref. Yes)												
No	-0.136	0.737	0.854	0.873 (0.206,3.703)	0.153	0.429	0.722	1.165 (0.503,2.701)	-0.288	0.661	0.663	0.749 (0.205,2.738)
Willingness to be admitted to nursing homes (Ref. Yes)												
No	1.143	0.719	0.112	3.135 (0.766,12.840)	0.906	0.437	**0.038**	2.475 (1.050,5.834)	0.236	0.642	0.713	1.267 (0.360,4.458)
Number of chronic diseases (Ref. ≥2)												
1	-1.638	0.825	**0.047**	0.194 (0.039,0.980)	0.266	0.550	0.629	1.304 (0.444,3.835)	-1.904	0.708	**0.007**	0.149 (0.037,0.596)
0	-2.421	0.867	**0.005**	0.089 (0.016,0.486)	-0.325	0.489	0.506	0.722 (0.277,1.884)	-2.096	0.800	**0.009**	0.123 (0.026,0.590)
Number of children (Ref. ≥2)												
1	1.032	0.968	0.287	2.806 (0.421,18.719)	0.963	0.730	0.187	2.621 (0.627,10.956)	0.068	0.826	0.934	1.071 (0.212,5.401)
0	-3.079	1.203	**0.011**	0.046 (0.004,0.487)	-0.900	0.685	0.189	0.406 (0.106,1.556)	-2.178	1.111	0.050	0.113 (0.013,0.998)
ADL (Ref. Moderate to severe dependence )												
Mild dependence	-1.449	1.087	0.182	0.235 (0.028,1.976)	1.130	0.929	0.223	3.097 (0.502,19.115)	-2.580	0.859	**0.003**	0.076 (0.014,0.408)
Independence	-3.704	1.197	**0.002**	0.025 (0.002,0.257)	0.520	0.895	0.561	1.682 (0.291,9.720)	-4.224	1.100	**< 0.001**	0.015 (0.002,0.127)
Social isolation (Ref. Yes)												
No	-0.884	0.943	0.348	0.413 (0.065,2.620)	-1.386	0.416	**0.001**	0.250 (0.111,0.566)	0.502	0.930	0.590	1.651 (0.267,10.216)

*B* Unstandardized Coefficient, *SE* Standard Error, *CI* Confidence Interval, *Profile 1* “most negative” group, *Profile 2* “moderately negative” group, *Profile 3* “positive” group

Compared to the “positive” group, those in the “moderately negative” group were significantly more likely to have no pension, to have been admitted to the nursing home involuntarily, and to be socially isolated, while the “positive” group was primarily characterized by a younger age (60–74 years, with ≥ 90 as reference). 

Compared to the “moderately negative” group, older adults in the “most negative” group were more likely to: be aged 75–89 years (ref. ≥90) and have no pension. Conversely, the “moderately negative” group was primarily characterized by having no comorbidities (ref. ≥2) and exhibiting only mild dependence or independence in ADL (ref. moderate to severe dependence).

### Profile differences and correlations

As shown in Table 4, statistically significant differences were observed among the three latent profiles of attitudes toward ageing for adults regarding self-efficacy and across all four domains of QoL (physical health, psychological health, social relationships, and environmental health). Attitudes toward ageing, self-efficacy, and the four dimensions of QoL conform to a normal distribution, so Pearson correlation analysis was adopted. The results (see supplementary materials Table S1) show that attitudes toward ageing, self-efficacy, and the four dimensions of QoL correlate.

**Table 4:** Effect of the different latent profiles of attitudes toward ageing on self-efficacy and four dimensions of qualtiy of life

Variables	Profile 1	Profile 2	Profile 3	Overall test	
	Mean (SE)	Mean (SE)	Mean (SE)	BCH χ2	*P*
Self-efficacy	18.677 (0.710)	23.609 (0.289)	28.331 (0.581)	114.204	< 0.001
Physical health	10.703 (0.391)	14.237 (0.137)	16.425 (0.187)	203.560	< 0.001
Psychological health	11.469 (0.234)	13.383 (0.079)	15.441 (0.147)	248.736	< 0.001
Social relationships	11.620 (0.310)	12.639 (0.144)	14.079 (0.303)	33.292	< 0.001
Environmental health	12.697 (0.188)	14.389 (0.097)	15.405 (0.181)	109.884	< 0.001

*Profile 1* “most negative” group, *Profile 2 *“moderately negative” group, *Profile 3 *“positive” group

### Mediation analysis of self-efficacy based on LPA

The four dimensions of QoL were analyzed by single factor analysis (see supplementary materials Table S2–S5), and variables with *p* < 0.05 were incorporated into the mediation model of the four dimensions of QoL as covariables, respectively. The mediation model with physical health as the outcome variable was adjusted for the following covariates: age, sex, pension, willingness to be admitted to nursing homes, number of chronic diseases, ADL, and social isolation. The mediation model with psychological health as the outcome variable was adjusted for the following covariates: age, sex, pension, insurance, willingness to be admitted to nursing homes, number of chronic diseases, number of children, ADL, and social isolation. The mediation model with social relationship as the outcome variable was adjusted for the following covariates: sex, marital status, pension, insurance, number of children, ADL, and social isolation. The mediation model with environmental health as the outcome variable was adjusted for the following covariates: sex, pension, insurance, number of chronic diseases, number of children, ADL, and social isolation.

Table 5 and supplementary materials Fig. 2A-D show the path coefficient of the mediating model. Take Profile 1 as a reference. Self-efficacy has 0.429 and 0.915 mediating effects between Profile 2 and Profile 3 and physical health, respectively, and the 95% Bootstrap confidence intervals are (0.201, 0.689) and (0.507, 1.406). It does not contain “0”, indicating that the mediation effect is significant. In addition, self-efficacy is also mediated between attitudes toward ageing profiles and the psychological health and environmental health dimensions of QoL. For social relationships, the bootstrap confidence intervals for the indirect effects included zero (Profile 2: 95% CI [-0.025, 0.323]; Profile 3: 95% CI [-0.053, 0.678]), indicating that the mediation effects were not statistically significant.

**Table 5:** The mediating effect of self-efficacy between attitudes toward ageing and qualtiy of life

	Path	Effect	SE	LLCI	ULCI
Outcome variable: Physical health					
Indirect effect	a2*b	0.429	0.125	0.201	0.686
	a3*b	0.915	0.230	0.507	1.406
Direct effect	c’ 2	1.562	0.279	1.013	2.111
	c’ 3	2.476	0.387	1.713	3.238
Total effect	c2	1.991	0.285	1.429	2.553
	c3	3.391	0.377	2.649	4.133
Outcome variable: Psychological health					
Indirect effect	a2*b	0.245	0.078	0.109	0.408
	a3*b	0.521	0.141	0.267	0.819
Direct effect	c’ 2	0.893	0.181	0.537	1.249
	c’ 3	2.099	0.251	1.606	2.592
Total effect	c2	1.138	0.183	0.778	1.498
	c3	2.620	0.241	2.145	3.095
Outcome variable: Social relationships					
Indirect effect	a2*b	0.140	0.089	−0.025	0.323
	a3*b	0.303	0.187	−0.053	0.678
Direct effect	c’2	0.545	0.292	−0.030	1.119
	c’3	1.090	0.407	0.289	1.890
Total effect	c2	0.685	0.282	0.130	1.240
	c3	1.393	0.370	0.665	2.121
Outcome variable: Environmental health					
Indirect effect	a2*b	0.144	0.060	0.039	0.276
	a3*b	0.330	0.125	0.099	0.596
Direct effect	c’ 2	1.136	0.200	0.743	1.529
	c’ 3	1.634	0.279	1.085	2.183
Total effect	c2	1.280	0.197	0.893	1.668
	c3	1.964	0.261	1.451	2.477

In the mediating model with physical health as the outcome variable, compared with Profile 1, the mediating effect ratio of Profile 2 was 21.547% (a2*b2/c2% = 0.429/1.991), and that of Profile 3 was 26.983%. In the mediating model with psychological health as the outcome variable, compared with Profile 1, the mediating effect ratio of Profile 2 was 21.529%, and that of Profile 3 was 19.885%. In the mediating model with environmental health as the outcome variable, compared with Profile 1, the mediating effect ratio of Profile 2 was11.250%, and that of Profile 3 was 16.802%.

## Discussion

The results of this study showed that the mean score of attitudes toward ageing of older adults was 70.053, which was lower than the previous research on older adults in nursing homes [16]. It may reflect a correlation with the fact that the study’s subjects are older adults in the “early transition period”. The new older adults in nursing homes encounter obstacles such as role change, habit adjustment, and interpersonal reconstruction [44]. Older adults are prone to psychological and physiological maladaptations when they just enter a new environment [9], which may have a negative impact on their attitudes toward ageing.

This may also indicate that such findings are linked to variations in how older adults perceive ageing across different countries and cultures. Under the influence of traditional Chinese culture, moving into a nursing home is typically regarded as a helpless choice made by older adults who have lost the capacity for self-care, and they are thus prone to developing negative attitudes toward ageing, viewing themselves as “old and useless” [9]. This serves as a reminder to focus more on older adults during the “early transition period” while addressing the difficulties associated with ageing.

### The analysis of the dimensions of attitudes toward ageing based on LPA

Based on LPA, the attitudes toward ageing of older adults in the “early transition period” were divided into three profiles: “most negative”, “moderately negative”, and “positive”. More than half of older adults fell into the “moderately negative” profile. Older adults with an AAQ total score of 72 or above are considered to hold a positive attitude toward ageing. Because each sub-scale contains the same number of items, a mean sub-scale score of 24 or above (the theoretical midpoint, not clinical thresholds) can be interpreted as a relatively positive attitude on that dimension. In our study, “positive” group scored higher than “moderately negative” group across all dimensions, and “moderately negative” group scored higher than “most negative” group. Besides, “most negative” group participants obtained average scores below 24 on all three dimensions, whereas “moderately negative” group and “positive” group participants scored below 24 only on psychological growth. Therefore, future interventions should simultaneously address psychosocial loss, physical change, and psychological growth—especially psychosocial loss—among older adults who share the demographic characteristics of “most negative” group, whereas those similar to “moderately negative” group and “positive” group can be primarily targeted for improving their attitudes toward psychosocial loss.

The physical change dimension emphasizes the positive experiences of health, physical exercise, and ageing itself. Embodied cognition theory suggests that physiological experiences activate mental states [45]. This indicates that degradation in one’s physiology may activate the psychological experience of negative physical change dimension. Older adults in the “early transition period” have not fully accepted nursing homes, and compared to those who have adapted, they are more likely to believe that a decline in their physiological functions forced them to leave their homes, which ultimately leads to an increase in the negative psychological experience of the physical change dimension. This dimension is the most crucial in distinguishing the three profiles, and is the focus of the intervention. Physical activity can improve older adults’ physical health and is considered an important intervention to improve their overall well-being [46]. Therefore, fitness facilities can be provided at the institutional level, and targeted exercise programs can be formulated according to the specific situation of older adults to encourage them to take physical exercise, improve their health status, enhance their happiness, and promote the formation of “old and useful”.

Psychosocial loss refers to the negative psychological and social experiences experienced by older adults [13]. Chinese people exhibit a “differential mode of association” in interpersonal interactions [47]. The “differential mode of association”, a concept describing the social structure of traditional China, takes the individual as the center and extends outward like ripples in water to form a social network with distinct tiers of closeness and distance [47]. Blood ties serve as the core bond of this structure, where people define the hierarchy of relationships and the boundaries of rights and obligations based on the proximity of kinship [47]. The existence of the “differential mode of association” causes Chinese older adults to pay more attention to social networks based on blood links and less independent than in Western societies, which are dominated by the “organizational mode of association” [47]. Older adults in the “early transition period” are faced with abrupt changes in their surrounding environment and spatial isolation from better-connected relatives and friends, leading to breaks in the original social network and maladaptation in psychological and interpersonal social relations, ultimately increasing the number of negative experiences in terms of psychosocial loss. Therefore, older adults should be actively assisted in rebuilding their interpersonal network, allowing them to reap more positive advantages from the new interpersonal network and, eventually, enhance attitudes toward ageing on the psychosocial loss dimension.

Psychological growth can be summarized as “wisdom” and “growth” in old age [13]. Centered on “filial piety”, the traditional Chinese family culture embodies the nation’s unique moral tenets: parents shoulder the responsibility of giving birth to and raising their children, and in return, children are obligated to show filial respect to their parents [48]. This is traditionally regarded as a natural “reciprocal care cycle” within a family. The ideal way to fulfill this duty is to have parents live with their family and be cared for personally by their loved ones, a practice that is bound up with love, honor and moral obligation [48]. In this cultural context, it is plausible that older adults tend to view moving into nursing homes as being deserted by their children for their lack of worth in old age [9, 16]. The decline of self-worth will lead to negative ageing attitudes [16]. Thus, one possible pathway is that a reduced sense of self-worth prevents these older adults from recognizing the “wisdom” and “growth” associated with the notion of “ageing is beneficial”, thereby reducing their positive psychological growth experiences related to ageing. To address this, interventions could focus on supporting older adults in rebuilding a sense of self-worth, teach them that rising age represents an increase in experience, which is also a valuable wealth. Recalling writing tasks can effectively activate self-worth and indirectly improve ageing attitudes [16]. Future research could further test this interpretive pathway by directly measuring constructs such as filial expectations, perceived family obligation, and self-worth within similar cultural settings.

### Influencing factors of attitudes toward ageing

#### Demographic characteristics

Comparisons between “most negative” group and “moderately negative” group revealed that individuals aged 75–89 years held more negative attitudes toward ageing than those aged 90 years or older. In contrast, comparisons between “moderately negative” group and “positive” group indicated that individuals aged 90 years or older had more negative attitudes than those aged 60–74 years. This means that the correlation between age and attitudes toward ageing is not a straight line. However, earlier research has shown that attitudes toward ageing gradually deteriorated with age [49]. Attitudes toward ageing do not completely deteriorate with chronological age (the actual length of time lived since birth), which may suggest that they are related to the existence of subjective age. Subjective age is the subjective assessment of an individual’s actual age [50]. It can reflect how older adults recognize their ageing. The more they realize they are becoming older, the more negative their attitudes toward ageing will become [51].

Second, older adults with no pension were found to be more likely to belong to the group with relatively more negative attitudes toward ageing, consistent with earlier research [52]. Older adults with pensions are more likely to stay in nursing homes with better environmental facilities and more abundant activities [53], making it easier for older adults in the “early transition period” to adapt to the new living environment and obtain more positive experiences, thus promoting the formation of positive attitudes toward ageing.

Furthermore, comparisons between “most negative” group and “positive” group revealed that older adults with two or more children were more likely to exhibit negative attitudes toward ageing than those with no children. Previous research has indicated that a higher number of children does not necessarily enhance intergenerational support for older adults; instead, having multiple children may lead to shared responsibility diffusing or even shifting among them, resulting in neglect of parental care needs [54]. Additionally, older adults with multiple children may have higher expectations of support from their offspring [54, 55]. This discrepancy between expectations and reality can foster feelings of failure and negative self-perception, such as, “I raised so many children, yet I still ended up in a nursing home,” which may subsequently contribute to more negative attitudes toward ageing.

#### Physiological factors

Older adults with chronic disease counts ≥ 2 were more likely to belong to the more negative attitudes toward ageing profile than those with chronic disease counts 0 or 1. Physical health has a significant impact on attitudes toward ageing, as earlier study has shown [56]. We postulate that this disease accumulation may trigger a “psychological stacking effect.” As an individual’s condition progresses from a single disease to multiple comorbidities, it does not merely represent a linear addition of physical burdens. Instead, the symptoms, treatments, and lifestyle constraints associated with various diseases interact synergistically, continuously reinforcing core negative perceptions of a “loss of bodily control” and a “diminished health identity.” This persistent signaling of physical decline leads older adults to internalize their experience, forming a pervasive and often irreversible negative self-identity that equates ageing directly with frailty. Consequently, future intervention programs should specifically target and prioritize older adults living with comorbidities.

Furthermore, older adults with moderate to severe dependence exhibited more negative attitudes toward ageing than those who were complete independence or mild dependence. Older adults with moderate-to-severe dependence in ADL experience a loss of independent living capacity, which often leads to an absence of meaningful social roles and subsequently fosters a sense of worthlessness. When a conflict arises between their self-perception as an “independent individual” and their actual behavior of “being dependent on others,” they are likely to attribute this dissonance to old age. For older adults rated as having moderate to severe ADL dependence, the core principle for effectively enhancing their daily living capabilities is “task decomposition.” Specifically, caregivers should systematically break down complex activities, such as dressing and grooming, into a series of simple, manageable steps. During implementation, caregivers provide only the most essential assistance and encourage the patients to actively complete the final steps within the sequence that they are capable of.

#### Psychosocial factors

Older adults who are involuntarily admitted to nursing homes tend to exhibit more negative attitudes toward ageing. Traditional Chinese culture and customs may strongly shape older adults’ preference to age at home, and enjoyment of family life plays a role in promoting their physical and mental health [57]. In Chinese culture, the family often serves not only as a source of daily care but also as a fundamental anchor for older adults’ self-worth and social identity. Rooted in the traditional of “filial piety”, ageing at home is commonly perceived as a validation of being needed and relied upon by one’s children. This perception may directly translates into psychological reinforcement for healthy ageing. When older adults are involuntarily institutionalized, they may perceive it not merely as a change of residence but as an implicit loss of their valued role within the family. This could lead them to believe that they have become useless in old age and have been abandoned by their families. Future studies could further examine this pathway by directly measuring constructs such as perceived family role loss, filial expectation discrepancies, and their links to ageing attitudes among involuntarily institutionalized older adults.

In addition, older people with social isolation have more negative attitudes toward ageing. This is consistent with previous research [58]. The new interpersonal network of older adults newly admitted to the nursing home has not been established, and social isolation is more serious. Compared to older adults who have adapted to living in nursing homes, they are more likely to feel depressed and lost, and thus have more negative attitudes toward ageing [58].

Therefore, it is recommended that greater attention be paid to older adults who are involuntarily institutionalized and socially isolated. Interventions should prioritize addressing their sense of loss in family value to help them accept their current situation. Furthermore, by fostering new social support networks, we can enhance their sense of belonging within the institution, ultimately facilitating successful adaptation.

### The mediating effect of self-efficacy between attitudes toward ageing and QoL

There is a positive correlation among all dimensions of QoL, self-efficacy, and attitudes toward ageing. In order to further verify the mechanism of action between the three, this study built a mediation model. The cross-sectional mediation analyses indicated that the relationships between attitudes toward ageing, three domains of QoL (physical health, psychological health, and environmental health), and self-efficacy were consistent with a mediation model. It is worth noting that the mediating model in this study is a cross-sectional one; the results do not imply causality, and further longitudinal research is still required.

Embodied cognition theory holds that cognition is embodied while the body is embedded in the environment, and cognition, body, and environment are a dynamic unity [45]. This means that negative perceptions of ageing may impact the physical health, psychological health, and environmental health dimensions of QoL. Previous studies have shown that long-term exposure to negative attitudes toward ageing is considered a stressor, and older adults with negative attitudes toward ageing often show negative coping styles, which increases the risk of negative physiological reactions and thus has adverse effects on physical and physiological health [59, 60], ultimately resulting in a decline in physical and physiological dimensions of QoL. Cognition, body, and environment are all interdependent and dynamic. Ageing-related negative cognitive and psychosomatic reactions impact older adults’ environmental perception, further affecting their ability to adapt to the environment and ultimately leading to the decline of the environmental health dimension of QoL.

Self-efficacy, as a positive coping attitude, can play a positive role in the formation of attitudes toward ageing and the self-regulation process and promote the improvement of QoL. Just as Bandura explained the mechanism of self-efficacy, self-efficacy plays an important role in the process of self-regulation of emotional states [22]. Therefore, positive coping attitudes of older adults, such as self-efficacy, can be improved to buffer some of the negative effects of the negative attitudes toward ageing on physical health, physiological health, and environmental health, encouraging older adults to view ageing with a more positive and calm attitude and, ultimately, achieving successful ageing.

However, this study did not find the mediating effect between self-efficacy and QoL in the field of social relationships. This may stem from the specific characteristics of the study population. The participants were older adults newly admitted to a nursing home, a group whose existing social networks are often disrupted by the environmental transition, while new social connections have not yet been fully established. This could potentially explain the lack of a significant mediating pathway in the social relationship dimension. The establishment of a social relationship network cannot be done overnight. Therefore, self-efficacy, as a subjective coping attitude, can play a small role in improving the objectively existing social relationship separation of older adults newly admitted to the nursing home.

### Practical suggestions for nursing home staff

Based on the findings, the following intervention strategies are suggested for nursing home staff, with priority given to residents aged 75–89, without pensions, having two or more children, diagnosed with two or more chronic diseases, exhibiting moderate to severe dependence in ADL, and those who were admitted involuntarily or experience social isolation. This demographic is more susceptible to developing negative attitudes toward ageing. Suggested measures for older adults in this group may include:

On the hardware front, it may be beneficial to upgrade fitness facilities and create personalized exercise programs tailored to different health statuses. Psychologically, the strategy could addresses “family value restoration” through regular family events (e.g., intergenerational meals, story-sharing) and by guiding children to show support, mitigating feelings of “family abandonment.” For older adults whose children are not local, staff could provide support by facilitating regular video calls and creating exclusive WeChat groups, which serve as a platform for sharing weekly updates such as daily life photos and key health metrics. The use of reminiscence writing tasks may help older adults reconnect with their personal worth. Coupled with explaining the concept that “ageing is accompanied by an accumulation of experience,” this approach could help reduce the perception of being useless in later life. Socially, creating interest and mutual-aid groups could enable residents to quickly build in-facility relationships and alleviates social isolation.

Moreover, it may be possible to mitigate the negative impact on QoL by enhancing self-efficacy and improving negative attitudes toward ageing among older adults in nursing homes. This could be achieved through multidimensional interventions focused on rebuilding confidence through experience, cognition, skills, and emotional state. Specifically, this might involve breaking down tasks into manageable sub-goals based on individual abilities, such as organizing personal items independently or participating in simple crafts, to help them accumulate successful experiences through task completion. Organizing positive peer-sharing sessions or case studies can also help adjust cognition through role modeling. Furthermore, skill-based activities could be carried out, such as simple gardening, handcrafts, and painting. Additionally, assisting older adults in maintaining regular routines and balanced diets, organizing indoor exercise sessions, and incorporating relaxation techniques can alleviate anxiety, thereby providing comprehensive support for self-efficacy development.

### Limitation

This study has some limitations. First, as this is a cross-sectional study, longitudinal research is needed in the future to further examine the mediating role of self-efficacy between attitudes toward ageing and QoL. Second, the samples come from several nursing homes in Wenzhou, China, so it is necessary to conduct a multi-region and large-sample study to improve the representativeness and reliability of the study and make the research results more universal. Third, as all survey content was based on older adults’ self-reports, this may introduce self-report bias. Therefore, future studies should adopt more objective measures. Fourth, many other confounding factors such as depression were not controlled for in this study, and future research should consider incorporating more control variables. Fifth, as a data-driven method, LPA has inherently sample-specific results. Finally, the results of this study show that attitudes toward ageing does not completely change with the change of biological age, which may be related to the existence of subjective age. Subsequent relevant studies should comprehensively consider the influence of biological age and subjective age on attitudes toward ageing.

## Conclusions

Older adults during the “early transition period” had negative attitudes toward ageing. It may be related to the Chinese traditional interpersonal communication mode, family culture, and various maladaptive problems. Older adults who have two or more children, chronic diseases, no pension, moderate to severe dependency, involuntary admission to nursing homes, and social isolation are associated with more negative attitudes toward ageing. Mediation analysis reminds that self-efficacy can be used as intervention targets to improve the QoL.

## Supplementary Information

Supplementary Material 1.

## Data Availability

The datasets used and/or analyzed during the current study available from the corresponding author on reasonable request.
