# Supplementary material for: Latent profiles of attitudes toward ageing during the nursing home “early transition period” and its correlation with quality of life
Source: BMC Geriatr. 2026 Feb 7;26:330. doi: 10.1186/s12877-026-07007-7 (PMC12983644; doi:10.1186/s12877-026-07007-7)
Supplement: Supplementary file 1 — Supplementary Material 1. [file 12877_2026_7007_MOESM1_ESM.docx]

**Supplementary materials**


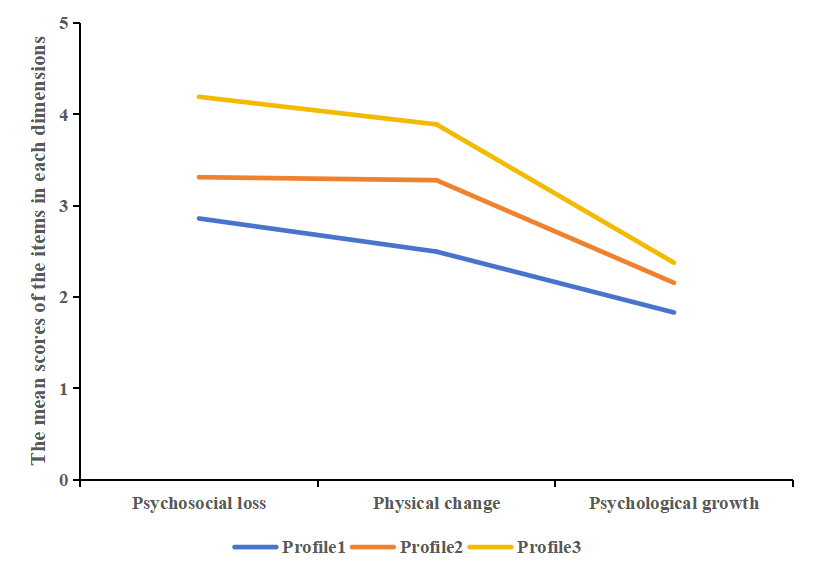


**Figure S1.** The mean scores of the items for all profiles on the AAQ.

**Note:** Profile 1 = “most negative” group, Profile 2 = “moderately negative” group, Profile 3 = “positive” group


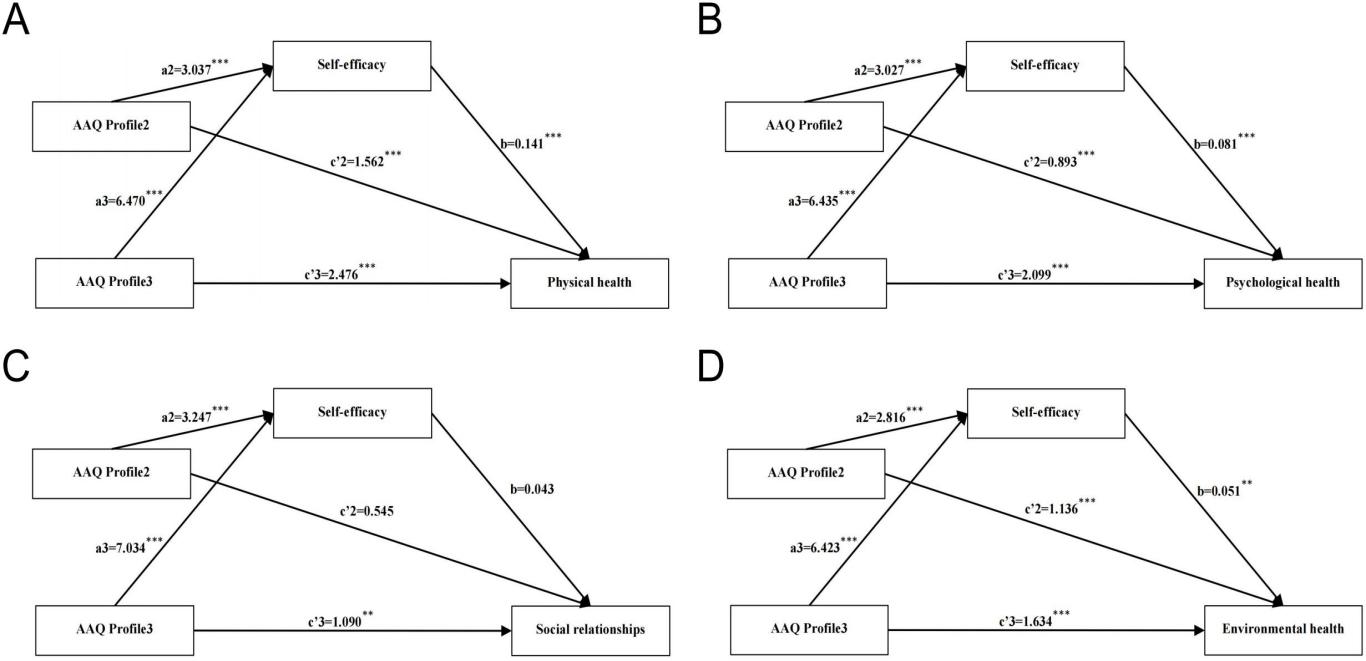


**Figure S2.** The path coefficient of the mediating model.

**Note:** **P＜0.01,***P＜0.001. All paths of the three mediating models, which take physical health, mental health and environmental health as outcome variables (Figure A, B, D), are statistically significant. This indicates that self-efficacy plays a mediating role in the relationship between aging attitudes and these three dimensions of quality of life. However, in the mediating model with social relationships as the outcome variable, the path between self-efficacy and social relationships is not statistically significant (Figure C).

**Table S1.** Descriptive statistics and correlation for attitudes toward ageing, self-efficacy and quality of life .

| **Variables** | **M(SD)** | **1** | **2** | **3** | **4** | **5** |
| --- | --- | --- | --- | --- | --- | --- |
| **1 Attitudes toward ageing** | 70.053(8.572) | 1.000 |  |  |  |  |
| **2 Self-efficacy** | 23.530(4.903) | 0.556^**^ | 1.000 |  |  |  |
| **3** **Physical health** | 13.975(2.517) | 0.671^**^ | 0.630^**^ | 1.000 |  |  |
| **4** **Psychological health** | 13.391(1.615) | 0.746^**^ | 0.618^**^ | 0.811^**^ | 1.000 |  |
| **5 Social relationships** | 12.702(2.118) | 0.361^**^ | 0.303^**^ | 0.295^**^ | 0.420^**^ | 1.000 |
| **6 Environmental health** | 14.258(1.499) | 0.523^**^ | 0.427^**^ | 0.494^**^ | 0.568^**^ | 0.655^**^ |

**Note:** **P＜0.01,***P＜0.001.

**Table S2.** Univariate analysis of physical health.

| **Variables** |  | **F/t** | **p** |
| --- | --- | --- | --- |
| **Age(years)** | 60-74 | 13.700^a^ | **＜0.001** |
|  | 75-89 |  |  |
|  | ≥90 |  |  |
| **Sex** | Male | 7.571^a^ | **0.006** |
|  | Female |  |  |
| **Marital status** | Married | 1.009^a^ | 0.318 |
|  | Widowed/Divorced/Single |  |  |
| **Educational level** | Primary school and below | 1.621 | 0.199 |
|  | Middle and high school |  |  |
|  | College and above |  |  |
| **Pension** | No | 25.412 | **＜0.001** |
|  | Yes |  |  |
| **Insurance** | No | 1.449 | 0.230 |
|  | Yes |  |  |
| **Willingness to be admitted to nursing homes** | No | 8.411 | **0.004** |
|  | Yes |  |  |
| **Number of chronic diseases** | 0 | 40.879^a^ | **＜0.001** |
|  | 1 |  |  |
|  | ≥2 |  |  |
| **Number of children** | 0 | 2.071 | 0.128 |
|  | 1 |  |  |
|  | ≥2 |  |  |
| **ADL** | Independence | 64.013^a^ | **＜0.001** |
|  | Mild dependence |  |  |
|  | Moderate to severe dependence |  |  |
| **Social isolation** | No | 15.856 | **＜0.001** |
|  | Yes |  |  |
| **Types of Nursing Homes** | Public-private | 0.581 | 0.562 |
|  | Private-private |  |  |

**Note:** a=Welch’s test, Variables with bolded p-values indicate significant effects and were included in the mediating model as controlled variables.

**Table S****3.** Univariate analysis of psychological health.

| **Variables** |  | **F/t** | **p** |
| --- | --- | --- | --- |
| **Age(years)** | 60-74 | 11.971^a^ | **<0.001** |
|  | 75-89 |  |  |
|  | ≥90 |  |  |
| **Sex** | Male | 13.764 | **<0.001** |
|  | Female |  |  |
| **Marital status** | Married | 0.040^a^ | 0.841 |
|  | Widowed/Divorced/Single |  |  |
| **Educational level** | Primary school and below | 0.586^a^ | 0.560 |
|  | Middle and high school |  |  |
|  | College and above |  |  |
| **Pension** | No | 31.104 | **<0.001** |
|  | Yes |  |  |
| **Insurance** | No | 7.008 | **0.009** |
|  | Yes |  |  |
| **Willingness to be admitted to nursing homes** | No | 16.050 | **<0.001** |
|  | Yes |  |  |
| **Number of chronic diseases** | 0 | 29.606 | **<0.001** |
|  | 1 |  |  |
|  | ≥2 |  |  |
| **Number of children** | 0 | 3.544 | **0.030** |
|  | 1 |  |  |
|  | ≥2 |  |  |
| **ADL** | Independence | 38.629 | **<0.001** |
|  | Mild dependence |  |  |
|  | Moderate to severe dependence |  |  |
| **Social isolation** | No | 31.571 | **<0.001** |
|  | Yes |  |  |
| **Types of Nursing Homes** | Public-private | 0.357 | 0.721 |
|  | Private-private |  |  |

**Note:** a=Welch’s test, Variables with bolded p-values indicate significant effects and were included in the mediating model as controlled variables.

**Table S4.** Univariate analysis of social relationship.

| **Variables** |  | **F** | **p** |
| --- | --- | --- | --- |
| **Age(years)** | 60-74 | 0.602 | 0.548 |
|  | 75-89 |  |  |
|  | ≥90 |  |  |
| **Sex** | Male | 11.947^a^ | **<0.001** |
|  | Female |  |  |
| **Marital status** | Married | 10.843 | **0.001** |
|  | Widowed/Divorced/Single |  |  |
| **Educational level** | Primary school and below | 1.474 | 0.231 |
|  | Middle and high school |  |  |
|  | College and above |  |  |
| **Pension** | No | 46.832 | **<0.001** |
|  | Yes |  |  |
| **Insurance** | No | 31.409 | **<0.001** |
|  | Yes |  |  |
| **Willingness to be admitted to nursing homes** | No | 0.059 | 0.808 |
|  | Yes |  |  |
| **Number of chronic diseases** | 0 | 0.526 | 0.591 |
|  | 1 |  |  |
|  | ≥2 |  |  |
| **Number of children** | 0 | 36.708 | **<0.001** |
|  | 1 |  |  |
|  | ≥2 |  |  |
| **ADL** | Independence | 4.928 | **0.008** |
|  | Mild dependence |  |  |
|  | Moderate to severe dependence |  |  |
| **Social isolation** | No | 26.507 | **<0.001** |
|  | Yes |  |  |
| **Types of Nursing Homes** | Public-private | -0.941 | 0.347 |
|  | Private-private |  |  |

**Note:** a=Welch’s test, Variables with bolded p-values indicate significant effects and were included in the mediating model as controlled variables.

**Table S5.** Univariate analysis of environmental health.

| **Variables** |  | **F** | **p** |
| --- | --- | --- | --- |
| **Age(years)** | 60-74 | 0.214 | 0.807 |
|  | 75-89 |  |  |
|  | ≥90 |  |  |
| **Sex** | Male | 6.684 | **0.010** |
|  | Female |  |  |
| **Marital status** | Married | 2.768 | 0.097 |
|  | Widowed/Divorced/Single |  |  |
| **Educational level** | Primary school and below | 2.767 | 0.064 |
|  | Middle and high school |  |  |
|  | College and above |  |  |
| **Pension** | No | 65.132 | **<0.001** |
|  | Yes |  |  |
| **Insurance** | No | 42.432 | **<0.001** |
|  | Yes |  |  |
| **Willingness to be admitted to nursing homes** | No | 2.466 | 0.117 |
|  | Yes |  |  |
| **Number of chronic diseases** | 0 | 7.330 | **<0.001** |
|  | 1 |  |  |
|  | ≥2 |  |  |
| **Number of children** | 0 | 9.396^a^ | **<0.001** |
|  | 1 |  |  |
|  | ≥2 |  |  |
| **ADL** | Independence | 9.960 | **<0.001** |
|  | Mild dependence |  |  |
|  | Moderate to severe dependence |  |  |
| **Social isolation** | No | 10.409 | **0.001** |
|  | Yes |  |  |
| **Types of Nursing Homes** | Public-private | 0.039 | 0.969 |
|  | Private-private |  |  |

**Note:** a=Welch’s test, Variables with bolded p-values indicate significant effects and were included in the mediating model as controlled variables.
